# Corticocerebellar White Matter Integrity Is Related to Naming Outcome in Post-Stroke Aphasia

**DOI:** 10.1162/nol_a_00107

**Published:** 2023-07-20

**Authors:** Zafer Keser, Erin L. Meier, Melissa D. Stockbridge, Bonnie L. Breining, Argye E. Hillis, Rajani Sebastian

**Affiliations:** Department of Neurology, Johns Hopkins University School of Medicine, Baltimore, MD, USA; Department of Neurology, Mayo Clinic, Rochester, MN, USA; Department of Communication Sciences and Disorders, Northeastern University, Boston, MA, USA; Department of Cognitive Science, Johns Hopkins University, Baltimore, MD, USA; Department of Physical Medicine and Rehabilitation, Johns Hopkins University School of Medicine, Baltimore, MD, USA

**Keywords:** aphasia, cerebellum, stroke, tractography

## Abstract

Studies have shown that the integrity of white matter tracts connecting different regions in the left cerebral hemisphere is important for aphasia recovery after stroke. However, the impact of the underlying structural connection between the cortex and the cerebellum in post-stroke aphasia is poorly understood. We studied the microstructural integrity of the cerebellum and the corticocerebellar connections and their role in picture naming. Fifty-six patients with left cerebral infarcts (sparing the cerebellum) underwent diffusion tensor imaging (DTI) and Boston Naming Test. We compared the fractional anisotropy (FA) and mean diffusivity (MD) values of the right and the left cerebellum (lobular gray and white matter structures) and cerebellocortical connections. Recursive feature elimination and Spearman correlation analyses were performed to evaluate the relationship between naming performance and the corticocerebellar connections. We found that the right, relative to left, cerebellar structures and their connections with the left cerebrum showed lower FA and higher MD values, both reflecting lower microstructural integrity. This trend was not observed in the healthy controls. Higher MD values of the right major cerebellar outflow tract were associated with poorer picture naming performance. Our study provides the first DTI data demonstrating the critical importance of ascending and descending corticocerebellar connections for naming outcomes after stroke.

## INTRODUCTION

Aphasia is a common and devastating consequence most frequently observed following left hemisphere cortical stroke. A growing literature points toward the importance of white matter tracts in understanding the neural mechanisms of language processing and determining the nature of language deficits and recovery patterns in aphasia (Ivanova et al., 2016). A large number of studies show that the integrity of white matter tracts in the lesioned left cerebral hemisphere is a crucial factor related to language outcomes after stroke (Bae et al., 2022; Bonilha et al., 2014; Ivanova et al., 2016; Kierońska et al., 2021). However, most investigations have focused on the cortical white matter tracts connecting the frontal, temporal, and parietal regions. There is limited information regarding the role of corticocerebellar white matter integrity in post-stroke aphasia. Assessing white matter networks that extend beyond the cortex may provide a more comprehensive assessment of brain damage and its impact on language processing.

In the past three decades, converging lines of research from clinical, neuroanatomical, and functional neuroimaging investigations have provided compelling evidence that the right cerebellum is engaged in a broad range of language and cognitive functions, well beyond its historical association with sensorimotor control (Mariën et al., 2014; Murdoch, 2010; Stoodley, 2012). In a large meta-analysis of functional magnetic resonance imaging (MRI) studies, consistent activations in the right cerebellar lobules were associated with language functions (Stoodley & Schmahmann, 2009). Damage to the right cerebellum has been associated with deficits in language tasks such as verbal fluency, naming, word generation, word stem completion, and syntactic processing (Baillieux et al., 2010; Gómez Beldarrain et al., 1997; Mariën et al., 2001; Richter et al., 2007). Cerebellar injuries due to various causes beyond ischemic stroke were also shown to be associated with language deficits in children and adults (Mariën & Borgatti, 2018). Neurodevelopmental abnormalities of frontocerebellar fibers were associated with deficits in language processing in children with autism or specific language impairments (Hodge et al., 2010). Similarly, patients with cerebellar vermian tumors had increased reading errors compared to healthy controls (Moretti et al., 2002).

The cerebellum is massively interconnected with the cerebral cortex, and neuroanatomical studies have shown bidirectional pathways between the cerebellum and multiple cortical structures involved in language and cognitive processing (Kelly & Strick, 2003; Schmahmann, 2001). Advances in diffusion-weighted MRI, including diffusion-tensor imaging (DTI), have helped to characterize the macro- and microstructural state of neuronal pathways between the cerebellum and cerebrum. DTI studies in healthy controls show that the cerebellum receives input from the cortex via the corticopontocerebellar (CPC) tracts and sends information to the cortex via the dentatorubrothalamocortical tract (DRTC; Keser et al., 2015).

A stroke in the cortex can disrupt the flow of information to the contralateral cerebellum and potentially cause a condition commonly termed crossed-cerebellar diaschisis (Feeney & Baron, 1986) consisting of cerebellar functional inactivation (Gold & Lauritzen, 2002), hypometabolism (Serrati et al., 1994), and hypoperfusion (Lin et al., 2009). Cortical strokes also can cause retrograde cerebellar changes in neuronal activity, metabolism, and structure (Förster et al., 2014; Keser et al., 2022). Studies investigating motor recovery in cortical strokes show that the structural integrity of corticocerebellar white matter tracts is linked to motor outcomes after stroke (Schulz et al., 2017; Wang et al., 2012). However, it remains largely unclear to what extent the structural integrity of the underlying corticocerebellar pathways is linked to language outcomes after stroke.

Our first aim was to explore the microstructural integrity of the relevant cerebellar structures and cerebellar connections to the cortex bilaterally using DTI in a cohort of stroke participants with infarct involving the left cerebral cortex. We hypothesized that the right cerebellar structures and tracts connecting the right cerebellum to the left cerebrum would show diminished microstructural integrity compared to their counterparts. The second aim was to investigate the association between naming and the microstructural integrity of cerebellar structures. We hypothesized that damage to the tracts connecting the right cerebellum and the left cerebrum would be associated with poorer naming performance. We focused on naming impairment, as this is the most common residual deficit in aphasia.

## MATERIALS AND METHODS

### Participants

The study was approved by the Institutional Review Board of the Johns Hopkins University School of Medicine, where all data collection occurred. All participants provided written informed consent.

This study included 56 right-handed (median age 60, range 29–81; 37 female), native English-speaking participants with infarct involving the left cerebral cortex. This study consisted of a retrospective analysis of prospectively collected data on participants with stroke involving the left cerebral cortex who had undergone language testing and MRI studies at the Stroke Cognitive Outcomes and Recovery (SCORE) Lab over a period of nine years (Table 1). We included participants who had both DTI and naming data. Additionally, we included 10 healthy right-handed participants (median age 55, age range 42–66; 5 females) from our center who underwent the same DTI study as normative data for right–left trends of the cerebellar structures and tracts.

**Table 1.** Summary of the demographics.

Subject	Age	Gender	Education (in years)	Boston Naming Test (/60)	Time since stroke (in weeks)
1	37	Male	12	8	24
2	64	Female	18	56	24
3	60	Female	14	46	24
4	60	Male	14	26	24
5	79	Female	12	52	24
6	68	Male	18	58	24
7	36	Female	12	36	24
8	54	Female	12	56	24
9	66	Male	14	0	24
10	74	Male	16	4	24
11	47	Female	15	58	24
12	74	Male	15	30	26
13	79	Male	22	34	36
14	29	Female	16	30	38
15	55	Female	15	58	47
16	36	Female	14	60	49
17	65	Female	14	58	50
18	78	Female	16	0	50
19	37	Male	14	60	50
20	29	Female	12	50	51
21	41	Female	18	58	51
22	64	Male	16	60	51
23	53	Male	18	24	52
24	50	Female	12	40	52
25	50	Male	16	48	52
26	41	Female	12	46	52
27	64	Male	16	26	52
28	63	Female	18	58	52
29	50	Male	16	56	52
30	47	Female	16	50	52
31	64	Male	18	58	52
32	65	Male	14	58	52
33	61	Male	12	58	52
34	29	Female	18	30	52
35	77	Female	16	60	54
36	50	Male	12	52	55
37	68	Male	10	36	56
38	55	Male	12	42	59
39	63	Female	18	58	60
40	62	Female	9	32	63
41	47	Male	18	10	64
42	79	Male	18	0	72
43	58	Male	15	20	83
44	81	Male	16	52	88
45	69	Female	12	12	104
46	65	Male	18	14	108
47	44	Male	16	32	112
48	52	Female	14	0	116
49	65	Male	10	0	116
50	79	Male	18	22	159
51	69	Male	12	32	171
52	54	Female	10	20	202
53	58	Male	16	10	216
54	69	Female	12	4	228
55	59	Male	13	20	472
56	72	Male	16	36	522

### MRI Acquisition and Preprocessing

3D sagittal acquired T1-weighted magnetization prepared rapid acquisition with gradient echo (MPRAGE) had a spatial resolution of 1 mm × 1 mm × 1 mm, field of view (FOV) = 256 mm × 256 mm × 180 mm, matrix size: 256 × 256, TR/TE: 2,000/3 ms. T2-weighted spin-echo images were acquired axially with a slice thickness of 2 mm with no gaps, FOV = 212 mm × 212 mm × 154 mm, matrix size: 256 × 256, TR/TE: 4,171/12 ms. FLAIR images were acquired axially with 2 mm slice thickness, FOV = 256 mm × 256 mm × 138 mm, matrix size: 256 × 256, TR/TE: 8,000/140 ms. Diffusion-weighted imaging (DWI) data were acquired axially with no gaps and a total of 32 diffusion orientations, with an additional b0 with TR/TE: 7,013/71 ms, FOV = 212 mm × 212 mm × 154 mm, matrix size: 256 × 256; b value = 700 s/mm^2^ (Landman et al., 2011), slice thickness/in-plane resolution = /2.2 mm/0.82 mm.

DWI data files were converted into NIfTI format using dcm2nii. We used DSI Studio for the remainder of the DWI pre-preprocessing. Diffusion-weighted images were resampled to 1 mm isotropic voxels, and the b-table was checked by an automatic quality control routine to ensure its accuracy (Schilling et al., 2019). Isotropic (1 mm × 1 mm × 1 mm) voxels were used in the analysis. Next, we generated indices of fractional anisotropy (FA; μ ± σ) and axial, radial, and mean diffusivities (MD; ×10^−3^ mm^2^ s^−1^) by tensor calculation.

### Lesion Load and Cerebellar Segmentation

**Figure 1.**
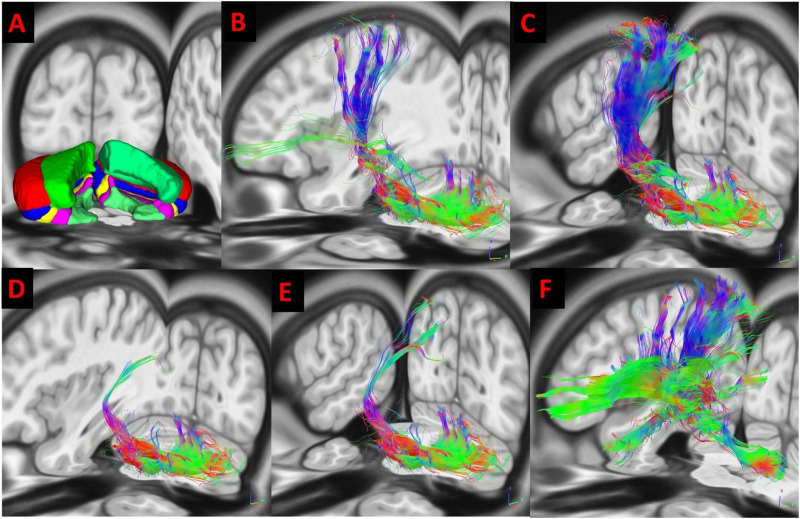
3D illustration of cerebellar segmentation on apparent diffusion coefficient maps from a single individual in the cohort. (A) Fronto-, (B) parieto-, (C) temporo-, (D) occipito-, and (E) ponto-cerebellar tracts, and (F) dentatorubrothalamocortical tract. Fibers are illustrated in color coded fashion (blue: fibers in superior inferior axis; red: fibers in medial lateral axis; green: fibers in anterior posterior axis). Magnetic resonance images of subject #16 were used for this illustration.

The lesion load was calculated as the percentage of total lesion volume to intracranial volume. Cerebellar segmentation was conducted by using probabilistic cerebellar SUIT atlas-based segmentation (a spatially unbiased atlas template of the cerebellum and brainstem; Diedrichsen et al., 2011; Diedrichsen & Zotow, 2015). Bilateral cerebellar segments VI, Crus I and II, VIIb, VIIIa, VIIIb, which previously were shown to be involved in language processing (Mariën & Borgatti, 2018) were extracted (Figure 1A). We included both gray and white matter tissue in the segmentation. Cerebrospinal fluid contamination was removed from the cerebellar lobules using the upper threshold radial diffusivity (RD) of 1.5 × 10^−3^ mm^2^ s^−1^ (Keser et al., 2021; Metzler-Baddeley et al., 2012). The regions then were edited with neuroanatomical guidance for each individual in native space (Diedrichsen et al., 2011; Diedrichsen & Zotow, 2015; i.e., moving the volume of interest in 3D space, removing neighboring tissues with smoothing and shaving functions).

### Tractography

**Figure 2.**
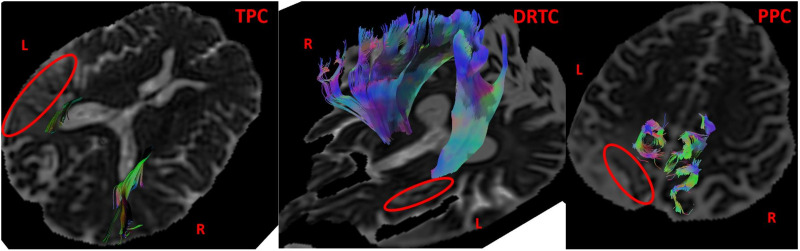
Illustration of temporopontocerebellar (TPC; from subject #4), dentatorubrothalamocortical tract (DRTC; from subject #26), parietopontocerebellar tracts (PPC; from subject #12) on the right (R) and left (L) in relation to the infarcts (circled with red). Please note loss of fibers in the infarcted tissue.

We utilized a semiautomated brute force and multiple regions-of-interest (ROIs) tractography method with fiber assignment by the continuous tracking (FACT) algorithm (Wakana et al., 2007). In the first step, DSI Studio automatic atlas-guided tractography recognition was performed to obtain middle and superior cerebellar pedunculi fibers; frontopontine, parietopontine, temporopontine, and occipitopontine tracts; and intracerebellar and thalamocortical fibers (Schilling et al., 2019). The FA threshold was 0.15, and the angular threshold was 70° (Keser et al., 2015). In step two, manual editing of the tracks was performed with neuroanatomy guidance (Keser et al., 2015), and the crossed fibers were combined. The right middle cerebellar peduncle and left corticopontine tracts were combined and vice versa to obtain the frontopontocerebellar (FPC; Figure 1B), parietopontocerebellar (PPC; Figure 1C), temporopontocerebellar (TPC; Figure 1D), occipitopontocerebellar (OCP; Figure 1E) tracts (Keser et al., 2015) bilaterally. These tracts connect the left cerebrum to the right cerebellum and the right cerebrum to the left cerebellum. For the DRTC tract (Figure 1F), the fibers arising from the ventrolateral, ventral anterior, intralaminar, and dorsomedial nuclei of the thalamus (Mariën & Borgatti, 2018) were isolated from the entire thalamocortical pathways and combined with the contralateral superior cerebellar peduncle. This tract connects the right cerebellum to the left cerebrum and the left cerebellum to the right cerebrum. Some of the tracts in relation to the lesions were also highlighted in different subjects (Figure 2). There were some visual overlaps between the tracts.

### Language Assessments

Stroke participants naming skills were assessed using the Boston Naming Test (BNT), a visual confrontation test for picture naming (Kaplan et al., 2001; Mack et al., 1992). The participants were asked to name line drawings of objects (30 or 60 items, all scaled to 60 items for analysis). Participants who scored 0 on the BNT were not excluded from the analysis. We focused on naming because anomia is present in all types of aphasia. In addition to the naming test, we administered an assessment of global language skills to all stroke participants. Due to the retrospective nature of the analysis, some participants completed the short form of the Boston Diagnostic Aphasia Examination, Third Edition (BDAE-3; Goodglass et al., 2001), and other participants completed Part 1 of the Western Aphasia Battery—Revised (WAB-R; Kertesz, 2007). Consistent with prior publications by our group (Keser et al., 2021; Meier et al., 2020), we captured global aphasia severity by first collating data from equivalent auditory comprehension, repetition, naming, and other verbal expression subtests from each assessment for each participant. Then, for each assessment (i.e., BDAE-3 or WAB-R), we generated subtest z scores using the formula *z* = (*x* − μ)/σ, such that *x* is a participant’s raw subtest score, μ corresponds to the sample’s subtest-specific mean score (averaged across all participants and time points), and σ corresponds to the sample’s subtest-specific standard deviation (Table 2).

**Table 2.** Summary of z scores from Boston Diagnostic Aphasia Examination-3 (BDAE-3) and Western Aphasia Battery (WAB-R) presented as mean and range.

BDAE-3 subtest	z-scores range (min–max)	WAB-R subtest	z-scores range (min–max)
Word comprehension	−2.53, 0.73	Auditory word recognition	−3.16, 0.33
Complex ideational material	−1.72, 1.02	Auditory verbal comprehension	−0.96, 0.96
Commands	−2.06, 0.78	Sequential commands	−2.58, 0.47
Repetition (words and sentences)	−2.01, 2.19	Repetition	−3.06, 0.52
Automatized sequences	−2.96, 0.48	Sentence completion	−3.18, 0.29
Responsive speech	−1.70, 1.43	Responsive speech	−3.18, 0.29
Total score	−2.14, 0.97	Total	−2.81, 0.45

### Statistical Analyses

The Shapiro–Wilk test was performed to test the normality of the variables. BNT, age, and many DTI metrics were found to be non-normal. For the first aim, the Mann–Whitney test was conducted to compare the right and left-sided FA and MD values of cerebellar regions and tracts. For the second aim, to account for correlated variables in high dimensional data (multi-collinearity), we performed recursive feature elimination (RFE) to determine the importance of DTI predictors for BNT. This model included age, education, time since stroke, and DTI metrics. Once the significant DTI predictors were identified, we conducted a Spearman correlation between BNT and significant predictors. Fisher Z transformation was performed to obtain Z values and 95% confidence intervals (CI). We controlled for multiple comparisons using false discovery rate (FDR) correction (*q* < 0.05). To show the correlation between the lesion load and the laterality of the cerebellar segments and tracts, we calculated the laterality index (LI) of MD of cerebellar segments/tracts using the following formula: (Left_segment/tract_[MD] − Right_segment/tract_[MD])/(Left_segment/tract_[MD] + Right_segment/tract_[MD]). We then used Spearman correlation to generate a correlation matrix using between lesion load and LIs of the cerebellar lobules/tracts. Similarly, a correlation matrix was created for other language scores and significant DTI predictors of BNT.

## RESULTS

The median BNT score was 36 [0–60]; education = 15 years (interquartile range (iqr): 4), lesion load = 0.61 (iqr: 2.64).

### Cerebellar Lobules

**Figure 3.**
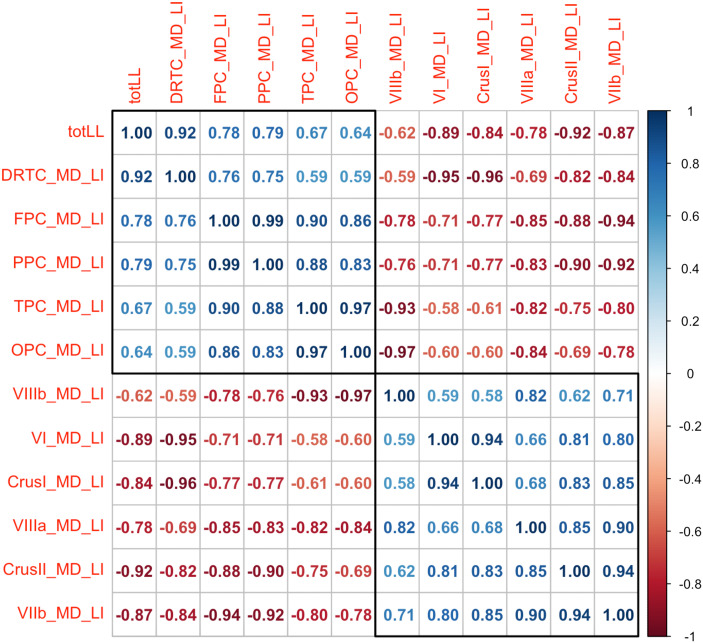
Heatmap correlation matrix between the laterality index (LI) of the cerebellar lobules/tracts and total lesion load (totLL). Of note, all the correlations in the heat map were significant (defined by *p* < 0.05). *Abbreviations*: DRTC = dentatorubrothalamocortical tract; FPC = frontopontocerebellar tracts; MD = mean diffusivity; PPC = parietopontocerebellar tracts; OPC = occipitopontocerebellar tracts; TPC = temporopontocerebellar tracts.

For participants with left cerebral infarcts, the FA value was significantly higher on the left compared to the right for the cerebellar segments VI (0.26(0.05) vs. 0.23(0.04), *w* = 2,139, *q* < 0.01), Crus I (0.23(0.03) vs. 0.21(0.03), *w* = 2,063 *q* < 0.01), Crus II (0.25(0.03) vs. 0.23(0.04), *w* = 2,124, *q* < 0.01), and VIIb (0.26(0.04) vs. 0.24(0.04), *w* = 1,924 *q* = 0.02). The MD values were lower for left compared to the right for the cerebellar segments VI (0.87(0.10) vs. 0.92(0.13), *w* = 1,041 *q* < 0.01), Crus I (0.90(0.10) vs. 0.96(0.12), *w* = 971, *q* < 0.01), and Crus II (0.82(0.08) vs. 0.85(0.12), *w* = 1,035, *q* < 0.01). No other contrast was statistically significant (Table 3). No comparisons were significant for the healthy participants (Table 4). Lesion load showed a significant negative correlation with LI of MD of all cerebellar lobules (compared to left cerebellum, lower microstructural integrity in the right cerebellar structures were associated with a higher lesion load; *p* < 0.05; Figure 3).

**Table 3.** Summary of comparison analyses in stroke patients.

	Left	Right	*w*	*p*	*q*	Cohen’s *d*
VI_FA	0.26 (0.05)	0.23 (0.04)	2139	<0.001	<0.001	0.66
Crus_I_FA*	0.23 (0.03)	0.21 (0.03)	2063	0.001	0.002	0.67
Crus_II_FA*	0.25 (0.03)	0.23 (0.04)	2124	<0.001	0.001	0.57
VIIb_FA*	0.26 (0.04)	0.24 (0.04)	1924	0.01	0.02	0.50
VIIIa_FA	0.27 (0.04)	0.25 (0.04)	1870	0.03	0.05	0.50
VIIIb_FA	0.28 (0.05)	0.27 (0.04)	1645	0.43	0.54	0.22
VI_MD*	0.87 (0.10)	0.92 (0.13)	1041	0.004	0.008	−0.43
Crus_I_MD*	0.90 (0.10)	0.96 (0.12)	971	0.001	0.002	−0.54
Crus_II_MD*	0.82 (0.08)	0.85 (0.12)	1035	0.004	0.008	−0.29
VII_MD	0.82 (0.10)	0.83 (0.10)	1249	0.12	0.17	−0.10
VIIIa_MD	0.82 (0.08)	0.82 (0.09)	1496	0.92	0.92	0.00
VIIIb_MD	0.84 (0.08)	0.84 (0.08)	1483	0.86	0.90	0.00
Intracerebellar_FA	0.26 (0.02)	0.26 (0.02)	1684	0.31	0.32	0.00
FPC_FA	0.49 (0.03)	0.49 (0.03)	1438	0.65	0.74	0.00
PPC_FA*	0.49 (0.04)	0.51 (0.03)	940	<0.001	0.002	−0.57
TPC_FA	0.47 (0.03)	0.48 (0.03)	1598	0.61	0.73	−0.33
OPC_FA	0.48 (0.02)	0.48 (0.03)	1447	0.69	0.75	0.00
DRTC_FA*	0.45 (0.03)	0.41 (0.03)	2380	<0.001	<0.001	1.33
Intracerebellar_MD*	0.84 (0.08)	0.87 (0.09)	1067	0.008	0.01	−0.35
FPC_MD*	0.93 (0.11)	0.88 (0.08)	2206	<0.001	<0.001	0.52
PPC_MD*	0.90 (0.11)	0.85 (0.07)	2295	<0.001	<0.001	0.54
TPC_MD*	0.96 (0.11)	0.91 (0.11)	1963	0.007	0.01	0.45
OPC_MD*	0.96 (0.12)	0.91 (0.09)	2003	0.004	0.008	0.47
DRTC_MD*	0.92 (0.09)	1.09 (0.11)	446	<0.001	<0.001	−1.69

*Note*. Significant results are highlighted in bold and with asterisk (*). *q* values were obtained from false discovery rate analyses. *w* values (sum of the positive or negative ranks) derive from Mann–Whitney test. *d* values were obtained from Cohen’s effect size analysis. *Abbreviations*: DRTC = dentatorubrothalamocortical tract; FA = fractional anisotropy; FPC = frontopontocerebellar tract; MD = mean diffusivity; OPC = occipitopontocerebellar tract; PPC = parietopontocerebellar tract; TPC = temporoparietopontine tract.

**Table 4.** Summary of comparison analyses for healthy subjects.

	Left	Right	*w*	*p*	Cohen’s *d*
VI_FA	0.29 (0.04)	0.27 (0.06)	68	0.19	0.39
Crus_I_FA	0.26 (0.05)	0.25 (0.03)	56	0.69	0.24
Crus_II_FA	0.27 (0.05)	0.26 (0.06)	55	0.74	0.18
VIIb_FA	0.26 (0.04)	0.27 (0.04)	45	0.74	−0.25
VIIIa_FA	0.27 (0.03)	0.27 (0.04)	47	0.85	0.00
VIIIb_FA	0.29 (0.05)	0.29 (0.04)	41	0.53	0.00
VI_MD	0.75 (0.07)	0.74 (0.05)	50	1.00	0.16
Crus_I_MD	0.78 (0.07)	0.79 (0.06)	33	0.22	−0.15
Crus_II_MD	0.82 (0.03)	0.82 (0.11)	47	0.85	0.00
VII_MD	0.82 (0.06)	0.82 (0.08)	54	0.8	0.00
VIIIa_MD	0.82 (0.08)	0.81 (0.15)	57	0.63	0.08
VIIIb_MD	0.77 (0.06)	0.76 (0.11)	58	0.58	0.11
Intracerebellar_FA	0.25 (0.01)	0.24 (0.01)	76	0.05	1.00
FPC_FA	0.5 (0.07)	0.51 (0.09)	50	1.00	−0.12
PPC_FA	0.5 (0.04)	0.49 (0.06)	50	1.00	0.20
TPC_FA	0.48 (0.02)	0.47 (0.03)	65	0.28	0.39
OPC_FA	0.49 (0.05)	0.49 (0.05)	50	1.00	0.00
DRTC_FA	0.49 (0.04)	0.48 (0.03)	63	0.35	0.28
Intracerebellar_MD	0.8 (0.02)	0.78 (0.02)	62	0.39	1.00
FPC_MD	0.81 (0.12)	0.79 (0.12)	57	0.63	0.17
PPC_MD	0.82 (0.12)	0.84 (0.11)	52	0.91	−0.17
TPC_MD	0.81 (0.11)	0.81 (0.11)	52	0.91	0.00
OPC_MD	0.82 (0.08)	0.84 (0.11)	54	0.80	−0.21
DRTC_MD	0.95 (0.19)	0.96 (0.19)	44	0.68	−0.05

*Note*. *w* values (sum of the positive or negative ranks) derive from Mann–Whitney test. *d* values were obtained from Cohen’s effect size analysis. *Abbreviations*: DRTC = dentatorubrothalamocortical tract; FA = fractional anisotropy; FPC = frontopontocerebellar tract; MD = mean diffusivity; OPC = occipitopontocerebellar tract; PPC = parietopontocerebellar tract; TPC = temporoparietopontine tract.

### Cerebellar Tracts

For participants with left cerebral infarcts, the FA value of PPC tract (0.49(0.04) vs. 0.51(0.03), *w* = 940, *q* < 0.01) was significantly lower on the left compared to the right. The left DRTC tract had significantly higher FA compared to the right (0.45(0.03) vs. 0.41(0.03), *w* = 2,380, *q* < 0.01). The MD values were significantly higher on the left for FPC (0.93(0.11) vs. 0.88(0.08), *w* = 2,206, *q* < 0.01), PPC (0.90(0.11) vs. 0.85(0.07), *w* = 2,295, *q* < 0.01), TPC (0.96(0.11) vs. 0.91(0.11), *w* = 1,963, *q* = 0.01), and OPC (0.96(0.12), 0.91(0.09), *w* = 2,003, *q* < 0.01), whereas the MD values were lower on the left for intracerebellar fibers (0.84(0.08) vs. 0.87(0.09), *w* = 1,067, *q* = 0.01), and DRTC tract (0.92(0.09) vs. 1.09 (0.11), *w* = 446, *q* < 0.01) (see Table 3). No comparisons were significant for the healthy participants (Table 4). Lesion load showed a significant positive correlation with LI (in comparison to contralateral counterparts, lower microstructural integrity in tracts connecting the right cerebellum to left cerebrum associated with a higher lesion load) of MD of all cerebellar tracts (*p* < 0.05; Figure 3).

### Correlation of Corticocerebellar White Matter Integrity With Naming

**Figure 4.**
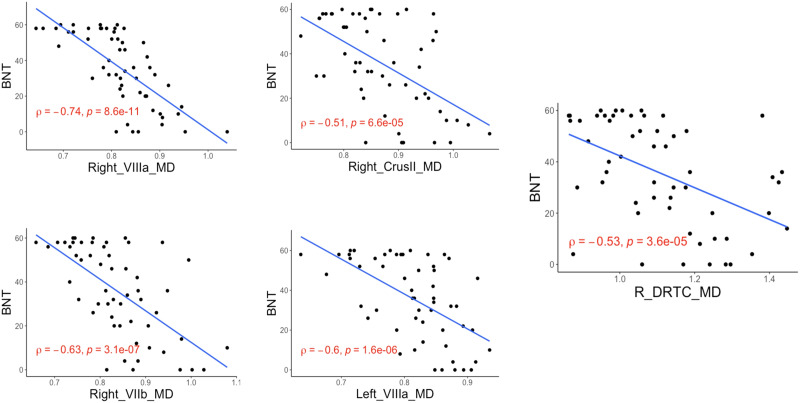
Scatterplot illustration of Boston Naming Test (BNT) and the mean diffusivity (MD) of right VIIIa, right VIIb, Crus II and left VIIIa segments as MD of right (R) dentatorubrothalamiccortical tract (DRTC) with rho and *p* values.

Based on RFE, the MD values of right DRTC tract, right VIIb, VIIIa, and Crus II segments, left VIIIa segments were significant predictors of naming accuracy. Out of the significant predictors, Spearman correlation analyses showed that the MD values of the right DRTC (rho = −0.53, Z = −0.59, 95% CI [−0.7, −0.31], *p* < 0.01), right VIIb (rho = −0.63, Z = −0.75, 95% CI [−0.77, −0.44], *p* < 0.01), right VIIIa (rho = −0.74, Z = −0.95, 95% CI [−0.84, −0.59], *p* < 0.01), right Crus II (rho = −0.51, Z = −0.56, 95% CI [−0.68, −0.29], *p* < 0.01), and left VIIIa (rho = −0.60, Z = −0.69, 95% CI [−0.75, −0.4], *p* < 0.01) segments showed significant correlation with BNT scores (Figure 4).

**Figure 5.**
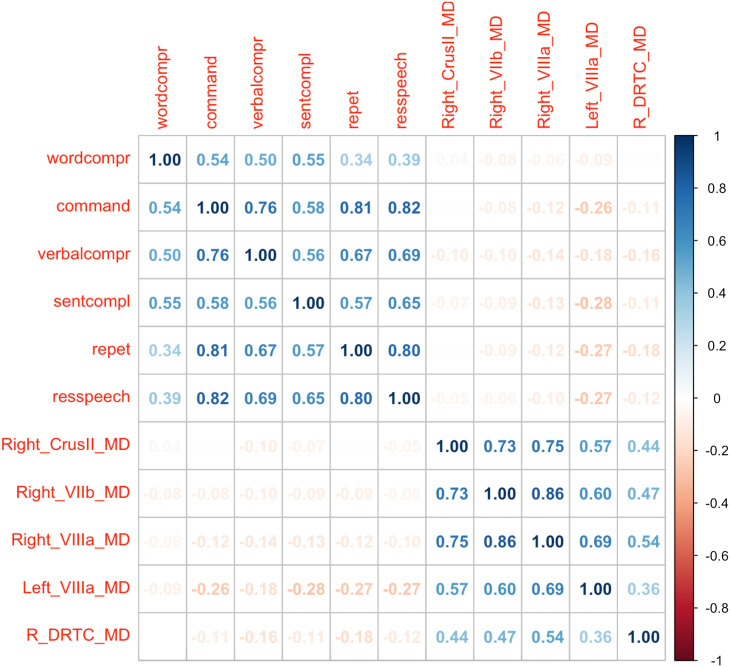
Heatmap correlation matrix of behavioral outcomes and regions that showed significant association with picture naming. Of note, none of the correlations in the heat map were significant (defined by *p* > 0.05). *Abbreviations*: R_DRTC = right dentatorubrothalamocortical tract; command = commands or sequential commands; repet = repetition of words and sentences; resspech = responsive speech; sentcompl = sentence completion; verbalcompr = verbal comprehension; wordcompr = word comprehension.

These regions did not show any significant correlation with other language scores (see Figure 5). Of note, time since stroke was also found to be a significant predictor, but this correlation was rather weak (rho = −0.28, Z = −0.29, 95% CI [−0.51, −0.018], *p* = 0.04).

## DISCUSSION

To investigate the role of the right cerebellum in language processing, we examined the anatomical connections between the cerebellum and the cerebrum using in vivo DTI tractography in post-stroke aphasia. Our results revealed that the right cerebellar segments and their connections to left cortical regions have reduced microstructural integrity after a left cerebral infarct. In addition, our results showed that picture naming was significantly associated with bilateral cerebellar nuclei and the right DRTC tract. This association was not observed for other language measures. These results have direct implications not only for a better understanding of the mechanisms associated with post-stroke language recovery but also for supporting the use of targeted interventions such as noninvasive brain stimulation techniques in post-stroke aphasia rehabilitation.

Stroke participants showed a significant MD increase for the left FPC, TPC, PPC, and OPC tracts compared to their homologous counterparts in the nonaffected cerebral hemisphere, whereas healthy controls did not show a difference in MD values for left versus right tracts. DTI analyses also showed a significant FA decrease and MD increase for the right cerebellar segments compared to the left cerebellar segments and the right DRTC tract compared to the left DRTC tract for stroke participants. This difference was not observed in the healthy controls. Reduced microstructural integrity in the ascending tracts from the right cerebellum to the left cortex suggests that cortical lesions trigger not only anterograde degeneration but also retrograde degeneration of axons that pass through the left cerebral stroke lesions (Schulz et al., 2017).

Reduction in the white matter integrity of the cerebellar language segments (left and right VIIIa) was associated with poorer naming performance (i.e., the lower the white matter integrity, the lower the naming accuracy). The integrity of the cerebellar outflow tract, the DRTC, also was positively associated with naming performance. We speculate that this could reflect the progressive structural alterations resulting from Wallerian degeneration in chronic stroke. Structural changes in Wallerian degeneration evolve over time with progressive loss of FA from the early subacute to the chronic stage (Thomalla et al., 2005). The strong correlation between lesion load and the microstructural integrity of nonlesioned parts of the cerebellar structures potentially supports the theory of secondary degeneration of cerebellar regions driven by lesion load.

Our results support the assertion that the reorganization of the spared brain regions beyond the lesion is crucial for post-stroke language recovery. There is growing evidence that the destruction of white matter and subsequent disruption of brain connectivity are strong predictors of post-stroke naming impairment (Bonilha et al., 2014; Meier et al., 2019). While this investigation focused on the contribution of feed-forward and feed-back cerebellar pathways to naming impairment, it complements existing evidence that implicates the more commonly investigated cortical tracts (Ivanova et al., 2016).

Previous studies also have substantiated the crucial role of the right cerebellum for language processing (Mariën et al., 2014; Murdoch, 2010). The results presented here support targeting the corticocerebellar network with interventional strategies based on neuromodulation with the goal of increasing the plasticity of the residual cortical language network and subsequently facilitating better post-stroke language outcomes in the chronic phase. Recent studies already have provided evidence that right cerebellar neuromodulation via transcranial direct current stimulation improves language skills in patients with chronic post-stroke aphasia (Marangolo et al., 2018; Sebastian et al., 2020).

In this study, picture naming correlated well with MD values due to a concomitant increase in axial diffusivity (AD) and RD, which affect MD. It was previously shown that ischemic infarcts lead to a downstream increase in diffusivity metrics such as MD, AD, and RD (Keser et al., 2021; Visser et al., 2019). Our finding aligns well with a previous longitudinal stroke study showing an increase in all the diffusivity measures (MD, AD, and RD) in perilesional ischemic tissue or demyelinating lesions (Klistorner et al., 2015; Umarova et al., 2017). Picture naming accuracy was not correlated with FA values.

Using the term “microstructural integrity” blindly without theoretical foundations might be misleading (Jones et al., 2013). However, when it comes to cerebral infarction, especially in the chronic stage, there are many diffusion studies supporting higher MD as a sign of white matter degradation (Keser, Meier, et al., 2020; Keser, Sebastian, et al., 2020). Although human postmortem studies are the gold standard to address these situations, consistent findings in human neuroimaging studies cannot be underestimated.

Our study has several limitations. First, our study design was cross-sectional; therefore, we were unable to investigate the longitudinal effects of change in cerebellar tracts and how that might relate to naming recovery. Second, we had a limited number of directions that prevented the usage of advanced diffusion methods, such as the constrained spherical deconvolution method. This could potentially be one of the reasons for the visual overlap of the tracts. Another reason could be the lack of high-resolution acquisition (<1 mm of voxel size) and low b values. Third, we did not have acute perfusion/cerebral blood flow imaging data in our participants, which meant we were unable to rule out the influence of crossed-cerebellar diaschisis. Fourth, as deterministic tracking failed to show decussating cerebellar fibers, our analysis generated cerebellar tracts in two segments and merged them to obtain the final tract. Finally, cerebellar structures were found to correlate only with the BNT score and not with the other language metrics (Figure 5). This selective association might be due to the cerebellum’s well-known role in the motor component of speech. Larger studies with more systematic language assessments are needed to elaborate further on this. Despite its limitations, our study is unique: To the best of our knowledge, this is the first example in the literature that provides evidence of secondary progressive degeneration in the right cerebellum and cerebellocortical connections after left cerebral infarction and associates this degeneration with poorer performance in picture naming. Our study may pave the way for targeted interventions such as noninvasive brain stimulation techniques that engage these spared pathways to prevent secondary degeneration and lead to better outcomes in post-stroke aphasia.

## FUNDING INFORMATION

Argye E. Hillis, National Institute on Deafness and Other Communication Disorders (https://dx.doi.org/10.13039/100000055), Award ID: R01 DC05375. Argye E. Hillis, National Institute on Deafness and Other Communication Disorders (https://dx.doi.org/10.13039/100000055), Award ID: R01 DC015466. Argye E. Hillis, National Institute on Deafness and Other Communication Disorders (https://dx.doi.org/10.13039/100000055), Award ID: R01 DC014664. Rajani Sebastian, National Institute on Deafness and Other Communication Disorders (https://dx.doi.org/10.13039/100000055), Award ID: R00 DC015554. Rajani Sebastian, National Institute on Deafness and Other Communication Disorders (https://dx.doi.org/10.13039/100000055), Award ID: R56 DC019639. Rajani Sebastian, National Institute on Deafness and Other Communication Disorders (https://dx.doi.org/10.13039/100000055), Award ID: R01 DC019639. Peter van Zijl, National Institutes of Health, Office of the Director (https://dx.doi.org/10.13039/100000052), Award ID: 1S10OD021648.

## AUTHOR CONTRIBUTIONS

**Zafer Keser**: Conceptualization; Formal analysis; Visualization; Writing – original draft; Writing – review & editing. **Erin L. Meier**: Formal analysis; Writing – review & editing. **Melissa D. Stockbridge**: Writing – review & editing. **Bonnie L. Breining**: Writing – review & editing. **Argye E. Hillis**: Conceptualization; Funding acquisition; Writing – review & editing. **Rajani Sebastian**: Conceptualization; Funding acquisition; Writing – review & editing.

## DATA AND CODE AVAILABILITY STATEMENTS

Data will be available upon reasonable request, such as reproducing the article’s key findings and conclusions or for building on the published work. MRI scans cannot be made openly available due to institutional restrictions. We used dcm2nii (https://www.nitrc.org/plugins/mwiki/index.php/dcm2nii:MainPage) and DSI Studio (version released 3/21/22) for the imaging analysis (https://dsi-studio.labsolver.org/). We used RStudio Version 4.1.3 (https://www.r-project.org/) for statistical analysis. Publicly available codes were used, and no extra in-house codes were created for the study.

## TECHNICAL TERMS

Fractional anisotropy: Quantifies the degree of anisotropic movement of water molecules.
Mean diffusivity: The average of the magnitudes of water molecule diffusion across all three axes.
Atlas-based segmentation: A method of bringing and registering previously labeled segments of the brain from a template into each individual subject’s brain.
